# Comparative Analysis of the Cytotoxic Effect of a Complex of Selenium Nanoparticles Doped with Sorafenib, “Naked” Selenium Nanoparticles, and Sorafenib on Human Hepatocyte Carcinoma HepG2 Cells

**DOI:** 10.3390/ijms23126641

**Published:** 2022-06-14

**Authors:** Elena G. Varlamova, Mikhail V. Goltyaev, Aleksander V. Simakin, Sergey V. Gudkov, Egor A. Turovsky

**Affiliations:** 1 Institute of Cell Biophysics of the Russian Academy of Sciences, Federal Research Center “Pushchino Scientific Center for Biological Research of the Russian Academy of Sciences”, 142290 Pushchino, Russia; goltayev@mail.ru; 2 Prokhorov General Physics Institute of the Russian Academy of Sciences, 38 Vavilove St., 119991 Moscow, Russia; avsimakin@gmail.com (A.V.S.); s_makariy@rambler.ru (S.V.G.)

**Keywords:** cancer, apoptosis, necrosis, selenium nanoparticles, sorafenib, selenium–sorafenib nanocomplex, gene expression, calcium signaling

## Abstract

Despite the use of sorafenib as one of the most effective drugs for the treatment of liver cancer, its significant limitations remain—poor solubility, the need to use high doses with the ensuing complications on healthy tissues and organs, and the formation of cell resistance to the drug. At the same time, there is more and more convincing evidence of the anticancer effect of selenium-containing compounds and nanoparticles. The aim of this work was to develop a selenium–sorafenib nanocomplex and study the molecular mechanisms of its anticancer effect on human hepatocyte carcinoma cells, where nanoselenium is not only a sorafenib transporter, but also an active compound. We have created a selenium–sorafenib nanocomplex based on selenium nanoparticles with size 100 nm. Using vitality tests, fluorescence microscopy, and PCR analysis, it was possible to show that selenium nanoparticles, both by themselves and doped with sorafenib, have a pronounced pro-apoptotic effect on HepG2 cells with an efficiency many times greater than that of sorafenib (So). “Naked” selenium nanoparticles (SeNPs) and the selenium–sorafenib nanocomplex (SeSo), already after 24 h of exposure, lead to the induction of the early stages of apoptosis with the transition to the later stages with an increase in the incubation time up to 48 h. At the same time, sorafenib, at the studied concentrations, began to exert a proapoptotic effect only after 48 h. Under the action of SeNPs and SeSo, both classical pathways of apoptosis induction and ER-stress-dependent pathways involving Ca^2+^ ions are activated. Thus, sorafenib did not cause the generation of Ca^2+^ signals by HepG2 cells, while SeNPs and SeSo led to the activation of the Ca^2+^ signaling system of cells. At the same time, the selenium–sorafenib nanocomplex turned out to be more effective in activating the Ca^2+^ signaling system of cells, inducing apoptosis and ER stress by an average of 20–25% compared to “naked” selenium nanoparticles. Our data on the mechanisms of action and the created nanocomplex are promising as a platform for the creation of highly selective and effective drugs with targeted delivery to tumors.

## 1. Introduction

Liver cancer is the second most common and fatal in the world after lung cancer, causing 745,000 deaths each year [1]. Treatment of patients with hepatocellular carcinoma diagnosed at an early stage is carried out by liver transplantation, but this method is acceptable only for 25% of patients [2], while the rest are exposed to radiation and chemotherapy, with all their inherent disadvantages and limitations. Chemotherapy as the main method of cancer therapy has practically not achieved breakthrough success in recent decades, due to the non-specific effects and significant damage to normal tissues during treatment. Treatment of patients with known anticancer drugs is complicated by hereditary or acquired resistance, which is accompanied by deterioration in the course of the disease and a decrease in patient survival [3].

Sorafenib is recommended by the U.S. Food and Drug Administration as a first-line drug for the treatment of liver and kidney cancer. It is a molecule with a high degree of targeting, the use of which effectively promotes the survival of cancer patients through the mechanisms of suppression of proliferation, angiogenesis, and induction of apoptosis [4]. However, despite its proven anti-cancer properties, sorafenib has a number of limitations in clinical use, among which the main one is its low bioavailability (8.4%) due to poor water solubility and its rapid metabolism, as well as dose-dependent side effects (skin toxicity, diarrhea, hypertension, and hand–foot syndrome [5,6,7]).

The development of nanotechnology makes it possible to develop approaches for the efficient transport of drug compounds selectively to cells and tissues in need of treatment. The use of nanoparticles as a carrier of the active compound is due to their small size and negative charge, which facilitates penetration through blood vessels and reduces non-specific binding. To date, individual nanocomplexes with sorafenib are known to activate anti-cancer signaling pathways more effectively than pure sorafenib. Such sorafenib delivery nanosystems include liposomes [8], solid lipid nanoparticles [9], polymeric nanoparticles [10] and micelles [11], gold nanoparticles [12], and inorganic nanoparticles [13]. The half-life of sorafenib in the body has been shown to be 8.8 h, while this figure for sorafenib-loaded micelles is 36.7 h, and the good physical stability of the micelles allows for a longer circulation of sorafenib in the body [13]. Sorafenib-loaded gold nanorods effectively suppress cancer cell proliferation upon tumor irradiation [14]. There is an approach of co-administration of sorafenib with other chemotherapy drugs. Doxorubicin, together with sorafenib, delivered to tumors in the form of nanoparticles based on PEG-b-cyclodextrin, exhibit a significantly more pronounced cytotoxic effect on HepG2 cells compared to their use alone [15,16].

Selenium nanoparticles (SeNPs) are of great interest for the development of therapeutic approaches to the treatment of various types of cancer due to their relatively simple synthesis, high biocompatibility, low toxicity, and ability to degrade in vivo, as well as significant radiosensitization effects with X-ray [17,18,19,20]. It is known that selenium nanoparticles are characterized by a pleiotropic effect, exerting an anticancer effect through the induction of apoptosis, but not affecting normal tissue cells [21] or dose-dependently suppressing apoptosis in brain cells during ischemia [22,23]. Therefore, in addition to delivering sorafenib, selenium nanoparticles themselves can enhance its effect by entering into cellular metabolism and activating anticancer signaling cascades. Of the currently known nanosystems based on nanoselenium and sorafenib, only one is known—PLGA-PEG-PLGA-based thermosensitive nanosystem, capable of controlled release of sorafenib and selenium nanoparticles. This nanosystem significantly reduced the expression of Ki67 and CD34, suppressing cell proliferation and tumor angiogenesis against the background of activation of the caspase3-dependent signaling cascade, inducing apoptosis [10]. However, PLGA-PEG-PLGA-based thermosensitive nanosystem works effectively in combination with chemotherapy, is temperature-controlled and expensive. Therefore, there is a need to develop new, more efficient sorafenib delivery nanosystems based on selenium nanoparticles and a comprehensive study of the molecular mechanisms of its anticancer effect, which was the key aim of this study.

## 2. Results

### 2.1. The Effect of Sorafenib, Selenium Nanoparticles, and Selenium–Sorafenib Nanocomplex on the Proliferation and Viability of HepG2 Cells

**Figure 1:**
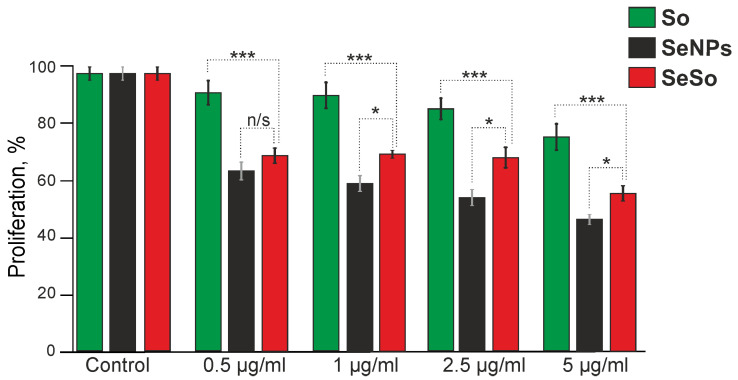
Proliferation assay of human hepatocarcinoma (HepG2) cells after 24-h exposure to various concentrations of sorafenib (So), selenium nanoparticles (SeNPs), and selenium–sorafenib nanocomplex (SeSo) performed by MTT assay. The optical density at 590 nm was measured, and the values of the respective untreated cells were defined as 100%. Mean values ± standard errors (SE) were determined by analysis of data from at least three independent experiments. Comparison of experimental groups regarding control, n/s—data not significant (*p* > 0.05), * *p* < 0.05, *** *p* < 0.001. n = 3.

The results of the MTT assay showed that a 24-h incubation of HepG2 cells with sorafenib resulted in a dose-dependent inhibition of cell proliferation by 9–22% (Figure 1, green columns), while treatment of cells for 24 h with SeNPs changed by 37–51% (Figure 1, black bars). The selenium–sorafenib nanocomplex also suppresses cell proliferation in a dose-dependent manner by 28–43% (Figure 1, red columns), which is significantly less effective than “naked” SeNPs. The SeSo nanocomplex is a selenium nanoparticle, on the surface of which sorafenib is sorbed. “Naked” nanoparticles seem to have a greater anti-proliferative activity, and to realize the synergistic effect of the drugs upon SeSo endocytosis, more time is required for the release of selenium inside the cell compared to SeNPs.

**Figure 2:**
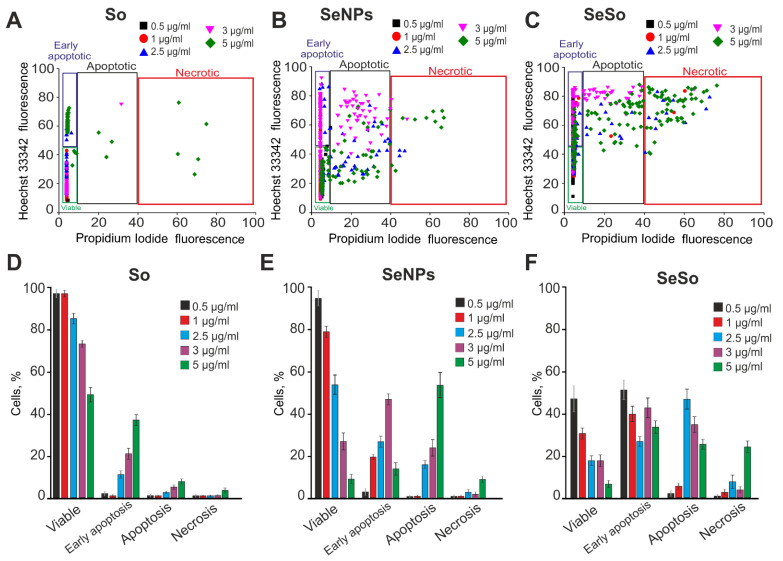
Pro-apoptotic effect of 24-h incubation of HepG2 cells with various concentrations of sorafenib (So), selenium nanoparticles (SeNPs), and selenium–sorafenib nanocomplex (SeSo). (**A**–**C**) X-axis: the intensity of PI fluorescence; Y-axis: the intensity of Hoechst 33342 fluorescence. Cells were stained with the probes after 24 h incubation with different concentrations of compounds. (**D–F**) Comparison of the effect of different concentrations of So (**D**), SeNPs (**E**), and SeSo (**F**) on HepG2 cell survival after 24 h incubation. Panel (**D**) is a calculation (technical replicate) of the data (mean ± SE) presented in panels (**A**–**C**), performed in 4 repetitions. n = 4. Cell images are shown in Supplementary Figures S1–S3.

In view of the fact that the processes of proliferation and apoptosis are regulated by various kinases and intracellular cascades, we conducted a comprehensive study of the cytotoxicity of So, SeNPs, and SeSo on the viability of HepG2 cells at 24 and 48 h exposure, after which the cells were stained simultaneously with Hoechst 33342 and Propidium Iodide. According to the obtained results, it can be concluded that after 24 h incubation of cells with So at concentrations of 2.5–5 µg/mL, early stages of apoptosis are induced in 18–39% of cells, while the presence of late stages of apoptosis was observed in 7–10% of cells when using So concentrations in the range of 3 and 5 µg/mL (Figure 2A,D; Supplementary Figure S1).

We have previously shown that SeNPs have an anticancer effect in various human tumor cell lines [21]. In this study, we studied the anticancer effects of SeNPs, 100 nm in size (Figure 9). 24 h after the addition of 1 µg/mL SeNPs to HepG2 cells, induction of early stages of apoptosis in 20% of cells was observed, and 3–5 µg/mL SeNPs induced late stages of apoptosis in 23–62% of cells, and a concentration of 5 µg/mL also caused necrosis in 12% of cells (Figure 2B,E; Supplementary Figure S2).

Nanoparticles in complex with So (SeSo) in 24 h contributed to the induction of early stages of apoptosis in 48% of cells already at a concentration of 0.5 μg/mL, and an increase in SeSo concentration to 2.5–3 μg/mL led to the induction of late stages of apoptosis in 38–43% of cells. Increasing the SeSo concentration to 5 μg/mL induced necrosis in 52% of the cells (Figure 2C,F; Supplementary Figure S3).

Therefore, after 24 h, sorafenib, starting at a concentration of 2.5 µg/mL, induced early stages of apoptosis in HepG2 cells, while SeNPs induced a similar effect already at concentrations of 1 µg/mL and 2.5 µg/mL, and higher concentrations induced late stages of apoptosis. The SeSo nanocomplex turned out to be more effective in inducing the death of HepG2 cancer cells, inducing early apoptosis already at 0.5 µg/mL, and late stages of apoptosis were similarly recorded with early apoptosis using the concentration range of 1–3 µg/mL. At the same time, as inhibitors of cell proliferation, SeNP and SeSo turned out to be quite effective even at low (0.5 μg/mL) concentrations, but “naked” SeNPs showed even higher efficiency.

**Figure 3:**
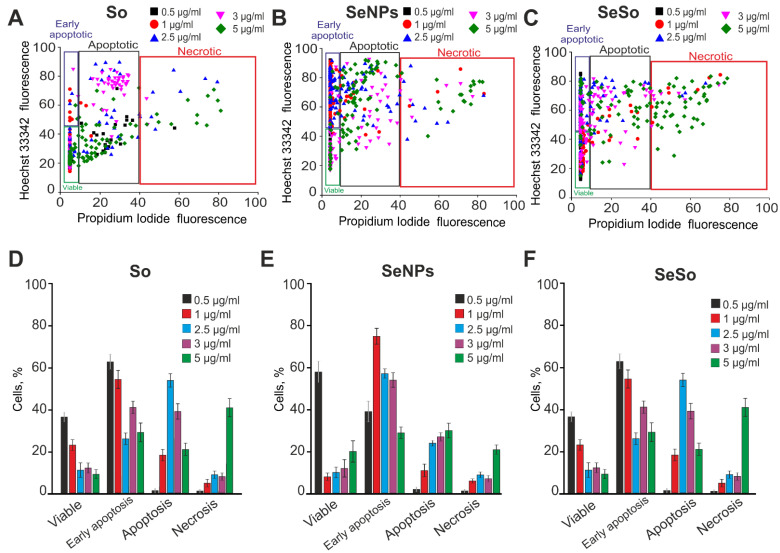
Pro-apoptotic effect of 48-h incubation of HepG2 cells with various concentrations of sorafenib (So), selenium nanoparticles (SeNPs), and selenium–sorafenib nanocomplex (SeSo). (**A**–**C**) X-axis: the intensity of PI fluorescence; Y-axis: the intensity of Hoechst 33342 fluorescence. Cells were stained with the probes after 48 h incubation with different concentrations of compounds. (**D**–**F**) Comparison of the effects of different concentrations of So (**D**), SeNPs (**E**), and SeSo (**F**) on HepG2 cell survival after 48 h incubation. Panel (**D**) is a calculation (technical replicate) of the data (mean ± SE) presented in panels (**A**–**C**), performed in 4 repetitions. n = 4. Cell images are presented in Supplementary Figures S4–S6.

Since the HepG2 line is more resistant to anticancer treatment compared to other cancer cell lines [24], the incubation time with the studied agents was increased to 48 h. Overall, 71% of cells had a decrease in the number of cells at the early stage of apoptosis when cells were treated with 2.5 µg/mL So (Figure 3A,D; Supplementary Figure S4). Interestingly, the late stages of apoptosis were recorded even at a So concentration of 1 µg/mL. In this case, necrosis was observed in 10–16% of cells at concentrations of 2.5 and 5 µg/mL So (Figure 3D).

After 48 h of incubation of cells with SeNPs (Figure 3B,E; Supplementary Figure S5) or SeSo (Figure 3C,F; Supplementary Figure S6), the process of cancer cell death was enhanced due to dose-dependent activation of apoptosis, but SeSo was still active in a lower concentration range compared to SeNPs.

**Figure 4:**
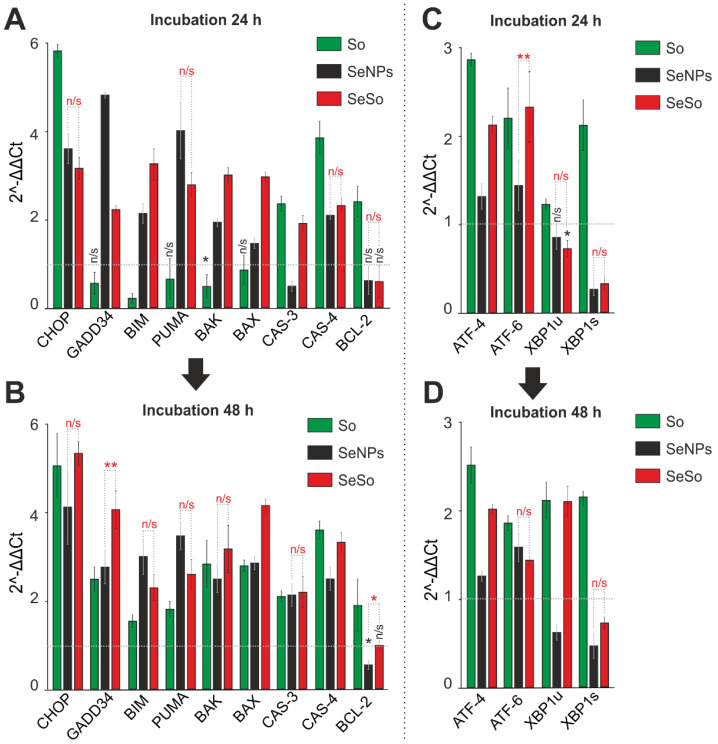
Expression analysis of genes encoding pro-apoptotic proteins (**A**,**B**) and ER-stress proteins (**C**,**D**) involved in apoptosis activation in HepG2 cells after 24 and 48-h incubation with 3 µg/mL of sorafenib (So), selenium nanoparticles (SeNPs), and selenium–sorafenib nanocomplex (SeSo). The level of expression in control cells was taken as 1. Statistical significance was assessed using paired t-test. Comparison of experimental groups with control is indicated by black asterisks. Unlabeled columns—significant values. n/s—data not significant (*p* > 0.05), * *p* < 0.05, ** *p* < 0.01. Significance comparisons between SeNPs and SeSo are indicated in red. n = 3.

When studying the expression patterns of a number of key pro-apoptotic genes, one can state an increase in the expression of their mRNA by two or more times in HepG2 cells after 24 h of exposure to selenium nanoparticles doped with sorafenib, as well as “naked” nanoparticles (Figure 4A). However, the pro-apoptotic effect of 24 h exposure to sorafenib turned out to be less pronounced, without significantly changing the expression of the GADD34, BAK, BAX, and PUMA genes, and in the case of the BIM gene, even a decrease in expression after 24 h of exposure was characteristic (Figure 4A). The effect of So on the expression of pro-apoptotic genes began to be registered only after 48 h of cell treatment, when an increase in the level of all the studied genes was observed (Figure 4C, green columns). An increase in the time of cell incubation with “naked” SeNP and SeSo contributed to the maintenance of the trend towards an increase in the expression of proapoptotic genes (Figure 4C).

It was important to study how the expression of well-known markers of adaptive and prolonged ER stress behaves, the activation of which is a common scenario when cancer cells are exposed to selenium-containing compounds of various nature, which has been repeatedly shown by us and other authors [25,26]. When evaluating the levels of mRNA expression of key markers of the three UPR pathways in HepG2 cells after 24 h of exposure to the studied agents, a change in the expression of mRNA of all studied transcription factors (*ATF-4*, *ATF-6*, and the spliced form of *XBP1*) was found, which may indicate activation pathways of apoptotic UPR, taking into account the increased expression of a number of pro-apoptotic genes. When cells were treated with sorafenib and selenium nanoparticles doped with sorafenib, an increase in the expression of *ATF-4* mRNA by more than 2–3 times was observed, which did not occur under the action of “naked” selenium nanoparticles. In addition, sorafenib caused an increase in the expression of the transcription factor *ATF-6* and the spliced form of the transcription factor *XBP1* by two or more times, while other agents, on the contrary, reduced the expression of the latter (Figure 4C). An increase in the cell incubation time up to 48 h led to the preservation of the expression trends of ER stress genes established for a 24-h exposure (Figure 4D). Thus, the experiments performed showed that activation of apoptosis in HepG2 cells requires high doses of So and 48 h of cell treatment, while “naked” SeNPs or the SeSo nanocomplex induce cancer cell death already after 24 h, and this process increases in proportion to the exposure time. At the same time, SeSo showed greater anti-cancer efficacy compared to “naked” SeNPs, since SeSo, added even at low concentrations, equally induces early and late stages of apoptosis already after 24 h, and SeNPs are predominantly early apoptosis, which can be reversible process. As for the expression of genes regulating apoptosis, using HepG2 cells as an example, significant differences in the expression of the studied genes were demonstrated when cells were exposed to sorafenib and selenium nanoparticles doped with sorafenib (SeSo) and without it (SeNPs). Interestingly, activation of apoptosis through ER stress occurred due to the effect of So on the activation of the UPR pathways (PERK, IRE1α, and ATF-6), while SeSo activated the PERK signaling pathway, as evidenced by a slight increase in the expression of the transcription factor *ATF-4*.

### 2.2. The Effect of Sorafenib, Selenium Nanoparticles, and Selenium–Sorafenib Nanocomplex on the Calcium Signaling System of HepG2 Cells

**Figure 5:**
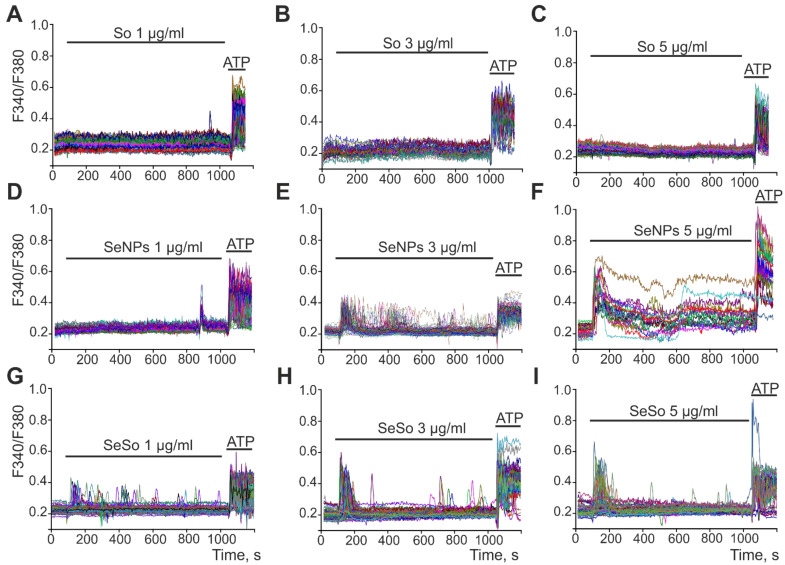
Registration of Ca^2+^-dynamics in HepG2 cells upon application of various concentrations of sorafenib (So, **A**–**C**), SeNPs (**D**–**F**), and SeSo (**G**–**I**). At the end of the experiment, 10 μM ATP was added. The figure shows the Ca^2+^-signals of individual cells. Number of parallel coverslips with cell cultures in each analysis = 4. n (number of cell culture passages) = 3.

The processes of activation of apoptosis and necrosis are closely associated with intracellular calcium signaling and can be activated by Ca^2+^ ions [27,28]. To record the dynamics of [Ca^2+^]_i_, the cells were loaded with a calcium-sensitive Fura-2 probe and, using fluorescence microscopy, the change in [Ca^2+^]_i_ was recorded upon application of So, SeNPs, and SeSo. Application of sorafenib at concentrations of 1, 3, 5 µg/mL to HepG2 cells did not cause the generation of Ca^2+^ signals (Figure 5A–C). At the same time, the calcium signaling system of cells was absolutely functional, and the addition of 10 μM ATP led to an increase in [Ca^2+^]_i_ through the activation of purinergic receptors and the mobilization of Ca^2+^ ions from the ER.

**Figure 6:**
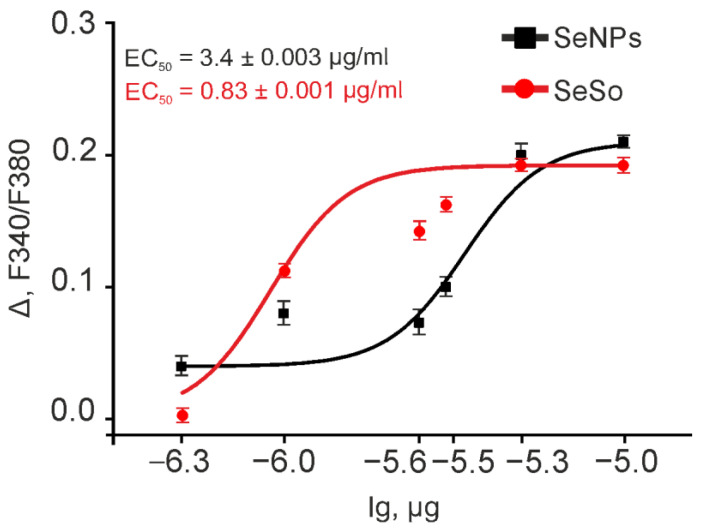
Dose-dependent change in the amplitude of Ca^2+^ signals in response to the application of SeNPs or SeSo to the HepG2 cells. Dependence of the amplitude of Ca^2+^ responses of HepG2 cells on the increase of SeNPs or SeSo concentration and its approximation by a sigmoid function. Each point represents the average value of the cell Ca^2+^-signal amplitude in one experiment ± SE. Cellular Ca^2+^-signals are shown in Figure 5. To plot dose dependences, we used the results of Ca^2+^-dynamic measurements on three independent cell cultures. Ca^2+^ signals for the application of all the concentrations shown in the figure are presented in Supplementary Figure S7. n (repetition for each point) = 6.

The application of 1 µg/mL SeNPs after 10–12 min caused the generation of Ca^2+^ signals in HepG2 cells (Figure 5D) in the form of a single pulse with the return of [Ca^2+^]_i_ to the basal level. Increasing the concentration of SeNPs to 3 µg/mL (Figure 4E) or 5 µg/mL (Figure 5F) led to rapid generation of Ca^2+^-responses by cells, diversity of Ca^2+^ signals (single Ca^2+^ pulses and Ca^2+^ oscillations), and dose-dependent growth signal amplitudes (Figure 6). At the same time, the addition of the same concentrations of SeSo induced rapid Ca^2+^ responses with different modes of oscillation already at a concentration of 1 µg/mL (Figure 5G). An increase in the SeSo concentration led to an increase in the amplitudes of Ca^2+^ signals, which were on average higher (Figure 6) compared to Ca^2+^ cell responses to the addition of SeNPs.

**Figure 7:**
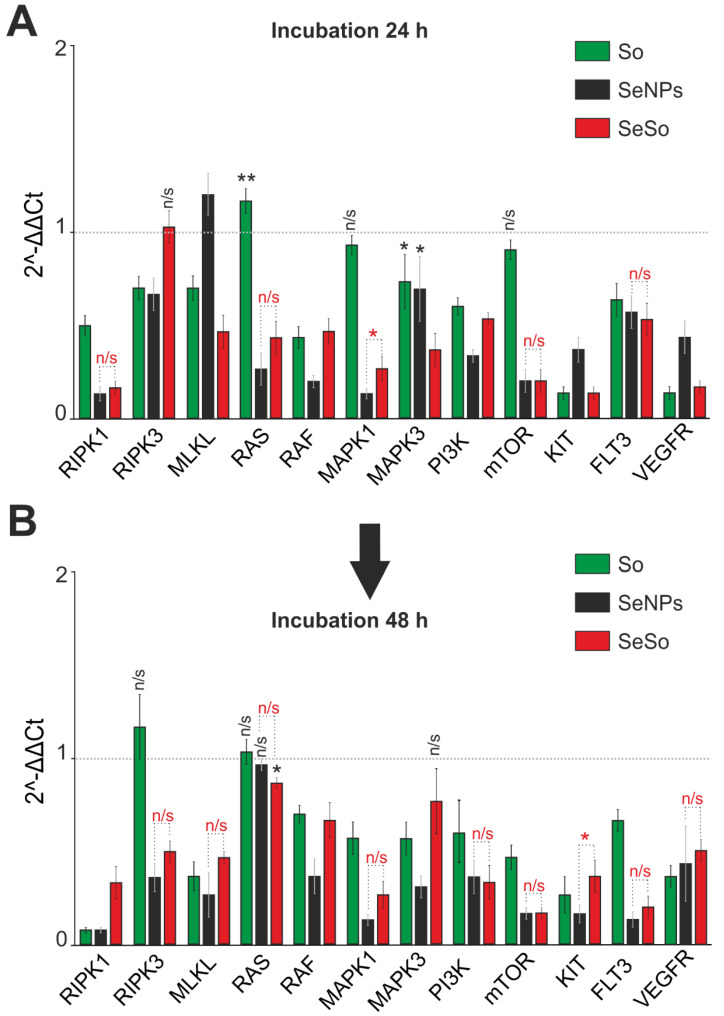
Analysis of the expression of genes encoding signal kinases in HepG2 cells after 24 (**A**) and 48 (**B**) hours of treatment with 3 µg/mL So, SeNPs, and SeSo. The level of expression in control cells was taken as 1. Statistical significance was assessed using paired *t*-test. Comparison of experimental groups with control is indicated by black asterisks. Unlabeled columns—significant values. n/s—data not significant (*p* > 0.05), * *p* < 0.05, ** *p* < 0.01. Significance comparisons between SeNPs and SeSo are indicated in red. n = 3.

As can be seen in Figure 7, the amplitudes of Ca^2+^ signals to the addition of SeSo in the concentration range of 1–3 µg/mL are, on average, significantly higher (Figure 6, red circles), compared to the application of “naked” SeSo (Figure 6, black squares). However, at high concentrations (5–10 µg/mL), the amplitudes of Ca^2+^-responses to SeSo and SeNPs do not differ significantly (Figure 6). Analysis of the dependence of the Ca^2+^ signal amplitude on the concentration of SeNPs and SeSo (Figure 6), reflecting the EC_50_ value, showed that SeSo is more effective in activating the calcium signaling system of HepG2 cells, since the EC_50_ for SeSo is 0.83 ± 0.001 µg/mL, and for “naked” SeNPs—3.4 ± 0.003 µg/mL.

Thus, sorafenib at the studied concentrations does not cause rapid reactions in the form of an increase in [Ca^2+^]_i_ in HepG2 cells, which may indicate the absence of a fast signaling component of sorafenib associated with intracellular calcium signaling, whereas SeNPs and SeSo induce a dose-dependent increase in [Ca^2+^]_i_ with the generation of various forms of Ca^2+^ signals. The selenium–sorafenib nanocomplex showed greater efficiency in activating the calcium signaling system of HepG2 cells compared to “naked” SeNPs.

### 2.3. Effects of Sorafenib, Selenium Nanoparticles, and Selenium–Sorafenib Nanocomplex on the Expression of Genes Encoding Signal Kinases and Selenium-Containing Proteins

According to the results of real-time PCR, treatment of HepG2 cells with sorafenib at a concentration of 3µg/mL for 24 h and 48 h (Figure 7) contributed to a decrease in the expression of a number of key kinases to one degree or another. Thus, a more than 2-fold decrease in the expression of serine-threonine protein kinases *RIPK1* and *RIPK3* was observed, as well as a decrease in the expression of mRNA of the pseudokinase *MLKL*. These data may indicate that sorafenib inhibited the activation of TNF-induced necroptosis. In addition, a decrease in the expression of *Raf* serine-threonine kinase, as well as *MAPK1* and *MAPK3* kinases, was observed after 48 h of treatment of HepG2 cells with So (Figure 7B), which may also indicate inhibition of the kinase cascade. Additional evidence for this can be reduced expression of the receptor tyrosine kinase *KIT* and *FLT3* (fms-like tyrosine kinase 3), which are also involved in the stimulation of the Ras-mediated signaling pathway, including *MAPK1* and *MAPK3*, even against the background of practically unchanged *Ras* expression (Figure 7). In addition, treatment of cells with So led to a significant decrease in *PI3K* kinase, which may indicate inhibition of the intracellular signaling pathway PI3K/AKT/mTOR, and, consequently, disruption of the cell cycle, reduced cell growth, and survival (Figure 7, green columns). The action of SeNPs and SeSo led to a significantly significant decrease in the expression of almost all the studied genes encoding the aforementioned kinases already after 24 h of exposure to HepG2 cells (Figure 7A), which turned out to be even more pronounced 48 h after their application to cells (Figure 7B). Thus, already 24 h after exposure of the cells to SeNPs and SeSo, the expression of mRNA of *RIPK1*, *MAPK1*, and *mTOR* kinases decreased by 5–10 times compared with the control.

**Figure 8:**
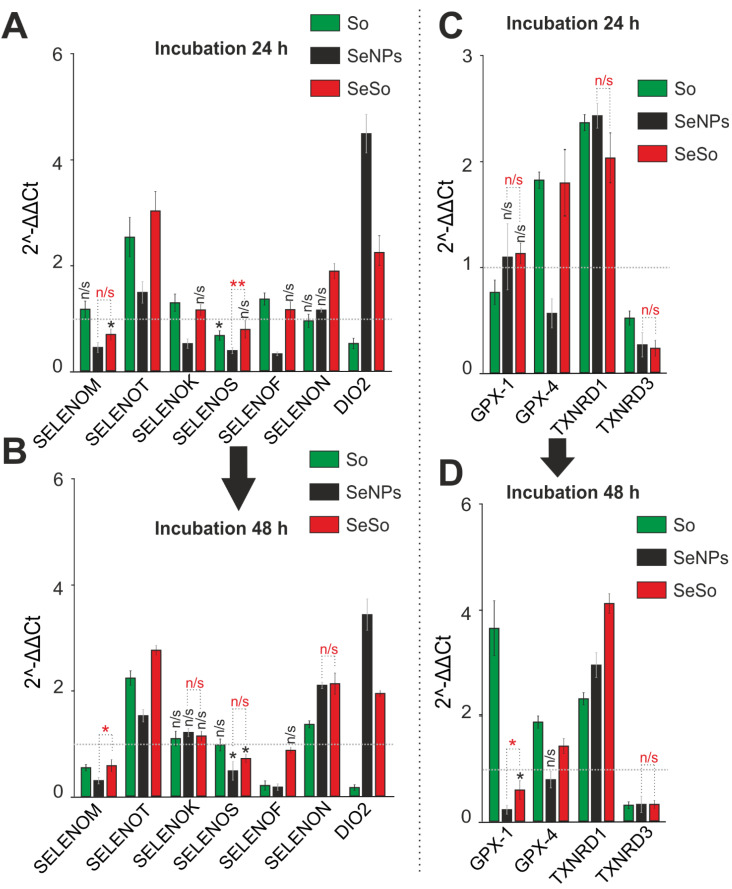
Analysis of the expression of genes encoding selenium-containing proteins in HepG2 cells after 24 (**A**,**C**) and 48 (**B**,**D**) hours of treatment with 3 µg/mL So, SeNPs, and SeSo. The level of expression in control cells was taken as 1. Statistical significance was assessed using paired *t*-test. Comparison of experimental groups with control is indicated by black asterisks. Unlabeled columns—significant values. n/s—data not significant (*p* > 0.05), * *p* < 0.05, ** *p* < 0.01. Significance comparisons between SeNPs and SeSo are indicated in red. n = 3.

Since selenium-containing agents were used in the study, it was extremely interesting to study the expression patterns of seven selenoproteins localized in the ER, the role of which in the regulation of ER stress was also proven in our previous works [29,30]. When evaluating the expression patterns of these genes, it can be noted that the nature of the change in their expression is ambiguous, and it is difficult to identify the patterns of its regulation depending on the active agent. Thus, already after 24 h of exposure to SeNPs cells, a decrease in *SELENOM* mRNA by almost 2 times was observed (Figure 8A); 48 h later, all studied agents also reduced the level of mRNA expression of this protein (Figure 8B). Additionally, a certain trend towards a decrease in mRNA expression was characteristic of the other two selenoproteins *SELENOS* and *SELENOF* under the action of SeNPs. At the same time, an almost 2-fold increase in *SELENOT* mRNA expression was observed under the action of So and its nanocomplex with selenium. A significant increase in the expression of mRNA encoding *SELENON* and *DIO2* was observed both 24 h (Figure 8A) and 48 h (Figure 8B) after treatment of cells with SeSo.

Analyzing the expression of mRNA of glutathione peroxidases *GPX1* and *GPX4*, as well as selenium-based thioredoxin reductases *TXNRD1* and *TXNRD3*, it can be concluded that treatment of this cell line with 3 µg/mL So showed a tendency to increase the expression of *GPX4* mRNA both after 24 h (Figure 8C) and after 48 h (Figure 8D) of exposure to cells, while *GPX1* was only after 48 h. In addition, there was a significant increase in *TXNRD1* mRNA expression caused by the action of all studied agents to one degree or another after 24 h and 48 h expression of *TXNRD3* mRNA in HepG2 cells, even after 24 h of cell treatment with all agents studied (Figure 8C,D).

**Figure 9:**
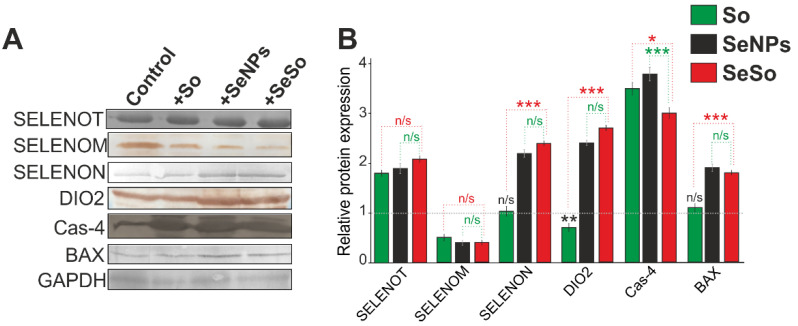
Western blot analysis of selenoproteins and pro-apoptotic proteins content in HepG2 cells after 48 h treatment with 3 µg/mL So, SeNPs, and SeSo. (**A**) Western blot analysis of protein content. (**B**) Quantification of proteins content in the samples. Comparison of treated groups vs Control (intact cells): *** *p* <0.001 (not marked). Comparison So-treated vs SeSo-treated experimental groups marked by red. SeNPs-treated vs SeSo-treated cell marked by green. n/s—data not significant (*p* > 0.05), * *p* < 0.05, ** *p* < 0.01, and *** *p* < 0.001. n = 3.

Based on Western blot results (Figure 9A; Supplementary Figure S8), real-time PCR data can be confirmed for a number of selenoproteins and pro-apoptotic markers. Thus, 48 h after application to the HepG2 cells So, SeNPs, or SeSo, a decrease in the expression of SELENOM and an increase in the expression of SELENOT, respectively, were observed. For the other two selenoproteins SELENON and DIO2, there was no change in their quantitative content in HepG2 cells after 48 h of So treatment and an increase in their number by more than 2 times after 48 h of treatment with SeNPs and SeSo (Figure 9B). Additionally, the results of Western blotting demonstrate an increase in the activity of the pro-apoptotic protein caspase-4 by more than 3 times compared with the control in cells treated with all the studied agents. In addition, an almost 2-fold increase in the content of the pro-apoptotic protein BAX, belonging to the Bcl-family, was observed after 48 h of exposure to cells SeNPs and SeSo, which may indicate the development of apoptosis along the mitochondrial pathway.

Thus, our results are consistent with previously obtained data on the decrease in the expression of a number of key intracellular kinases under the action of sorafenib [31,32], as well as a number of surface-located receptor tyrosine kinases (VEGFR1, KIT, FLT3) by two or more times compared to control cells. It can be concluded that the results of the analysis of the expression pattern of the studied genes make a significant contribution to explaining the greater resistance of cancer cells to the action of sorafenib compared to the action of nanoparticles.

## 3. Discussion

It is known that along with an effective anticancer effect on liver cells, sorafenib has a number of difficult-to-overcome limitations, the main one of which is its difficult absorption and the need to take high concentrations of the drug. As shown by the results of MTT assay, the use of concentrations of So 0.5–5 µg/mL does not cause a significant arrest of cell proliferation, which correlates with a relatively weak effect on the expression of cell cycle regulator kinases. However, the use of “naked” selenium nanoparticles, as well as those doped with sorafenib, led to inhibition of HepG2 cell proliferation and suppression of the expression of most kinases. So is known to cause cell cycle arrest through the regulation of a branched cascade of kinases, vascular endothelial growth factor receptor 2 (VEGFR-2) and platelet-derived growth factor receptor (PDGFR), and the STAT3 transcription factor [32,33]. The antitumor effect of So can also occur due to the inhibition of the RAF/MEK/ERK signaling cascade [31], which was also observed in our experiments, but with greater efficiency when using selenium nanoparticles. It has been shown on Huh7 hepatoma cells that the IC_50_ for sorafenib in the efficiency of cell cycle arrest is 15 ± 1.4 µM, and for SeNPs it is 5 ± 2 µg/mL, while doping of SeNPs with sorafenib leads to a decrease in IC_50_ to 820 ± 26 nM [34]. It was also shown on Huh7 cells that So in the effective concentration range of 15–30 μM causes a significant percentage of necrotic cell death (10.5–18%), while SeNPs at concentrations of 5–10 μg/mL are characterized by less pronounced necrosis (6–11%) [34]. In our experiments, it was found that SeSo practically suppresses necrotic processes in HepG2 cells, predominantly causing apoptosis, especially at its early stages, at lower concentrations (for SeNPs, 2.5 µg/mL, for SeSo, 0.5 µg/mL). It is known that MAP kinases are overexpressed in liver cancer [35], but the use of SeSo more effectively suppresses the expression of genes encoding proteins of the MAP kinase signaling cascade compared to sorafenib and SeNPs.

It is known that the action of sorafenib on liver cancer cells can induce robust expression of p53-upregulated-modulator-of-apoptosis (PUMA) [36], the expression level of which in our experiments was many times higher when SeNPs and SeSo were used. An increase in PUMA expression against the background of the absence of effects on the level of BCL-2 indicates the activation of the p53-independent apoptosis pathway through SIRT1, the release of cytochrome c from mitochondria, including due to increased oxidative stress [36,37,38]. Using vitality tests and pro-apoptotic gene expression analysis, it was also shown that So may trigger non-apoptotic cell death in regenerating livers by features resembling mitotic catastrophe [36]. At the same time, activation of caspase-3-induced apoptosis occurs only in hepatocytes with a cancerous phenotype. Despite the absence of caspase-3 activation in normal hepatocytes after sorafenib treatment, cell cycle inhibition, incorrect mitosis, and enhanced non-apoptotic liver injury during liver regeneration occur [36]. In our experiments, the SeNPs and SeSo developed by us, even after 48 h of exposure, caused necrotic cell death and inflammation in a small part of cancer cells, and then only when high doses were used. Thus, both variants of nanoparticles turned out to be the most effective in inducing apoptosis and suppressing necrosis compared to So, both due to suppression of the expression of *RIPK1*, *RIPK3*, *MLKL* kinases involved in the transition of apoptosis to necrotic process, and due to increased expression of a number of pro-apoptotic genes, ER stress markers, and selenoproteins. Thus, increased expression of a number of pro-apoptotic genes and, at the same time, a more noticeable inhibitory effect of expression was observed after 24 h of exposure to a number of enzymes of key kinase cascades, including *RAS*, *MAPK1*, *RAF*, *RIPK3*, and *mTOR* kinases. p53 is known as a tumor suppressor protein which is mutated in many cancer types involved in the induction of apoptosis.

In our experiments, activation of the Ca^2+^ signaling system of HepG2 cells under the action of selenium nanoparticles was established and doping of nanoparticles with sorafenib led to an increase in the amplitude of Ca^2+^ cell signals and a decrease in the EC_50_ value, which correlated with inhibition of cell proliferation and increased apoptosis. There is strong evidence of calcium-dependent suppression of cancer cell proliferation and migration by So, which occurs due to deactivation of FAK (focal adhesion kinase) and inhibition of Akt signaling. For example, the addition of lactate calcium salt to colorectal cancer cells causes an increase in [Ca^2+^]_i_, which correlates with the inhibition of the above signaling pathways [39]. The continuous influx of Ca^2+^ ions into the cytosol is one of the mechanisms of apoptosis activation due to calcium-dependent activation of proteolytic and catabolic degradation enzymes (proteases and phosphatases) [40]. In addition, there are data on calcium-dependent induction of ER stress under the action of So through the mobilization of cytoplasmic calcium, activation of PKR-like ER kinase (PERK), induction of IRE1α and XBP1 splicing, phosphorylation of eukaryotic translation initiation factor 2α (eIF2α), inhibition of protein translation, and induction of GADD153 and GADD34 [41], the level of which was also most effectively increased by SeNPs and SeSo even after 24 h of cell treatment. An increase in the expression of GADD family proteins enhances apoptosis in response to ER stress in various cell systems [42]. There is a suggestion that So causes powerful ER stress and ROS overproduction, which is not suppressed by antioxidants [41]. In our work, these effects were not studied, but we can assume an even more pronounced and stable effect of SeSo, even when using lower concentrations. It has been shown on cardiomyocytes that pre-incubation with high concentrations (10–30 μM) of So causes the release of Ca^2+^ ions from the sarcoplasmic reticulum and its depletion by more than 60%. This action of So suggests one of the ways through which it can disrupt the functioning of the cardiovascular system by suppressing contraction [43], however, in our experiments, many times lower concentrations of SeSo showed anticancer efficacy, which suggests that the negative effect of high doses is leveled. We also explored So on the cardiovascular system, although it requires additional studies in vivo. In our experiments, the application of So to HepG2 cancer cells did not cause an increase in [Ca^2+^]_i_ in acute experiments, while SeNPs and SeSo contributed to the rapid activation of the Ca^2+^ signaling system of these cells, probably leading to ER-stress-induced activation of apoptosis, since we previously showed the mechanism pro-apoptotic action of “naked” SeNPs through the activation of the phosphoinositide signaling cascade and the mobilization of Ca^2+^ ions from the ER [44]. There is evidence that So, at a concentration of 10 μM, causes an increase in [Ca^2+^]_i_ within 15 min after addition in U937, Jurkat cell lines, however, these are immune cell lines, and on other cell lines, including cancer cells, neither we nor other authors have obtained data on a rapid sorafenib-induced increase in [Ca^2+^]_i_. The same study showed that So does not lead to the generation of Ca^2+^ signals by K562 cells [41], although we do not rule out the effects of So on the Ca^2+^ system of HepG2 cells after 24 h and 48 h of exposure.

SeNPs and SeSo proved to be extremely effective in regulating the expression of ER stress proteins. It is known that PERK, activating transcription factor 6 (ATF6), and inositol-requiring enzyme 1 (IRE1) represent two ER transmembrane proteins that serve as ER stress sensors and mediate the UPR. Activation of IRE1 promotes X-box binding protein 1 (XBP1) mRNA splicing, an event that is required for translation of activated/spliced XBP1 [45,46]. Addition of 10 µM sorafenib to human leukemia U937, Jurkat, and K562 cells was found to result in rapid accumulation of IRE1α protein and more moderate XBP1 expression, similar to application of the well-known ER stress inducers, thapsigargin, or tunicamycin. Increasing the concentration of sorafenib to 30 μM leads to an even more pronounced increase in the expression of these proteins. In our experiments, SeSo activated ER stress, already at a concentration of 3 µg/mL, induced apoptosis even at 0.5 µg/mL without induction of necrosis, which demonstrates the high efficiency of the nanocomplex compared to sorafenib. Under conditions of ER stress, IRE1α, TRAF2, and ASK1 interact, which leads to the activation of apoptosis through JNK [47]. In our previous experiments, it was demonstrated that SeNPs, through the induction of ER stress, lead to the activation of apoptosis in various cancer cell lines [21]. In the presented series of experiments, doping of SeNPs with sorafenib leads to more pronounced ER stress and induction of apoptosis through the expression of ATF-4, ATF-6, XBP1u genes, and an increase in [Ca^2+^]_i_. Other proteins of ER stress-induced apoptosis are caspase-2 and caspase-4, whose expression levels increase with sorafenib incubation time (8 to 16 h), an effect similar to the addition of thapsigargin or tunicamycin [41,48,49]. Depletion of luminal ER calcium stores reflects ER stress and leads to UPR [50,51]. Under conditions of prolonged ER stress under the action of SeNPs and SeSo, protein folding is disrupted, and programmed cell death is activated through the activation of CHOP (C/EBP homologous protein) mediator, which is a transcription factor involved in the increase of the expression of a lot of genes related with apoptosis pathway [52,53]. Early stages of apoptosis under the action of SeNPs and especially SeSo are recorded after 24 h in our experiments and can be explained by the rapid activation of CHOP, as well as through ceramides and sphingolipids [54,55].

The liver is the main metabolic organ and maintains the energy homeostasis of the body through the regulation of the glucose cycle and its circulation in the blood [56]. Selenium and selenium-containing agents are essential micronutrients for liver function and, through the activation of AKT1/FOXO3a proteins, are involved in the regulation of carbohydrate and lipid metabolism in the liver [57]. Through SELENOF and AKT1/FOXO3a/PYGL, selenium-containing agents regulate glucose and lipid homeostasis in the liver, preventing metabolic disorders. High selenium intake (3.0 mg Se/kg; 10.0-fold of adequate Se) induces lipid accumulation in liver cells, activates lipogenesis, and reduces lipolysis and fatty acid oxidation [58]. Knockdown of SELENOF on a high-calorie diet leads to the development of steatosis [59]. In our experiments, SELENOF expression remained at the control level only when using the selenium–sorafenib nanocomplex, while sorafenib or SeNPs led to the suppression of its expression, which can be regarded as their negative effect. We have previously shown the anti-apoptotic effect of SELENOM and the pro-apoptotic effects of SELENOT [60]. In this study, a decrease in SELENOM mRNA expression was found after cell treatment with two types of nanoparticles, as well as an increase in SELENOT mRNA expression, especially after cell exposure to selenium nanoparticles in combination with sorafenib under conditions of increased expression of pro-apoptotic genes. A significant increase in DIO2 mRNA expression was also observed in HepG2 cells after their treatment with nanoparticles and, conversely, decreased when exposed to sorafenib. It is difficult to explain the specific role of this selenoprotein with the action of each of the compounds, but we observed a similar correlation between increased expression and apoptotic death of various cancer cells after exposure to selenium nanoparticles [21]. In addition, our results indicate a change in the redox status in cancer cells caused by the action of the studied agents, which is evidenced by a change in the expression of a number of key selenium-containing thioredoxin reductases and glutathione peroxidases. Despite the fact that earlier studies by other authors have repeatedly shown that inhibition of GPX1, GPX 4, and TXNRD1 contributed to increased oxidative stress in cancer cells [61,62,63,64], our results indicate an increase in their expression under the influence of the studied agents. Real-time PCR results for some of the studied selenoproteins and pro-apoptotic markers were confirmed by Western blotting in our experiments (Figure 9). In our case, such growth should probably be considered as a protective antioxidant response, which is insufficient under conditions of prolonged ER stress, sharp inhibition of key kinase pathways, and ultimately leads to apoptotic cell death to one degree or another, with apoptosis prevailing over necrosis, in the case of cell incubation with SeNPs and SeSo.

The concentrations of sorafenib used for the treatment of liver cancer are known to be 200–400 mg when taken two times a day. At the same time, serious negative side effects on the cardiovascular system, skin, kidneys, etc., are revealed [65,66]. In vitro studies in liver cancer cell lines have shown that sorafenib is effective in inducing apoptosis at concentrations of 10 mg/mL and above [67,68]. In our experiments, the selenium–sorafenib nanocomplex induced apoptosis at concentrations of 0.5 μg/mL, while at 5 μg/mL necrosis was recorded in HepG2 cell culture, and the use of higher doses was of no interest. Additionally, sorafenib at a concentration of 0.5 to 5 µg/mL had no effect on Ca2+ signaling, induction of apoptosis, and proliferation of HepG2 cells in our experiments. In the future, our results obtained in the in vitro model can be extrapolated to the in vivo model in order to confirm the more effective anticancer effect of sorafenib in the composition of the nanocomplex with selenium in order to reduce the dose of the active compound and level the side effects of high doses of sorafenib.

## 4. Materials and Methods

### 4.1. Reagents

Adenosine 5′-triphosphate sodium hydrate (ATP, A1852), DMEM (D6429), Hanks’ balanced salt solution (HBSS, H4641), and HEPES sodium salt (H7006) were purchased in Sigma-Aldrich, St. Louis, MO, USA; Hoechst 33342 (H1399), and propidium iodide (P1304MP) was purchased from Molecular Probes, Eugene, OR, USA; reverse transcriptase MMLV (SK022S), SYBR Green I PCR Master Mix (PK147L) was purchased from Evrogen, Moscow, Russia; Fura-2AM (cat. no. F1221), and fetal bovine serum (10099141) was purchased from Thermo Fisher Scientific, Waltham, MA, USA; Selenium and SeSo nanoparticles (courtesy of Dr. SV Gudkov, Prokhorov General Physics Institute, Russian Academy of Sciences, Moscow, Russia).

### 4.2. Preparation of Selenium Nanoparticles and Sorafenib-Doped Selenium Nanoparticles (SeSo)

**Figure 10:**
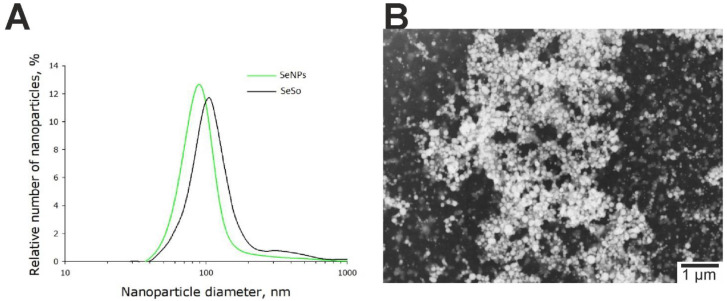
Size and morphology of selenium nanoparticles obtained by laser ablation and selenium–sorafenib noncomplex. (**A**) Evolution of the hydrodynamic diameter distribution of selenium nanoparticles before (SeNPs) and after sorption of sorafenib molecules (SeSo). (**B**) TEM micrographs of selenium nanoparticles preparation. Scale bar—1 μm.

Selenium nanoparticles were obtained by laser ablation in deionized water with a resistivity of 18 MΩcm. A solid target was placed at the bottom of the cuvette under a thin layer of water (no thicker than 2–3 mm). A solid target was irradiated with a laser beam through a thin layer of water (λ = 1064 nm; T = 4–200 ns; f = 20 kHz; P = 20 W; E_p_ = 1 mJ). The mixing of the laser beam on the target was carried out along a given trajectory in the form of parallel straight lines inscribed in a rectangle using an LScanH galvanomechanical scanner (Ateko-TM, Moscow, Russia) [69]. Depending on the characteristics of laser radiation, the speed and trajectory of the laser beam, it is possible to obtain colloidal solutions of selenium nanoparticles with specified geometric parameters. The size of nanoparticles was characterized using a DC24000 analytical centrifuge (CPS Instruments, Prairieville, LA, USA). Nanoparticle concentration and hydrodynamic radius were evaluated using Zetasizer Ultra Red Label (Malvern, UK) [70]. It was found that the resulting preparation of selenium nanoparticles has a monomodal size distribution (Figure 10). The average size of nanoparticles is about 100 nm, the half-width is in the range of 70–130 nm. The zeta potential of nanoparticles is about −30 mV. The morphology of nanoparticles was studied by a 200FE transmission electron microscope (Carl Zeiss Microscopy GmbH, Jena, Germany). According to electron microscopy data, nanoparticles have a spherical shape (Figure 10). The microscope is equipped with an attachment for energy-dispersive X-ray spectroscopy. The method of energy dispersive X-ray spectroscopy confirms that the nanoparticles consist of zero-valent selenium.

Sorafenib (Bayer HealthCare AG, Leverkusen, Germany) was applied to selenium nanoparticles as follows. A solution of sorafenib was prepared in citrate buffer (pH 4.1). The solubility of sorafenib in water is pH dependent. At high pH, the solubility of sorafenib decreases. Selenium nanoparticles were added to the sorafenib solution and incubated for 30 min. After that, centrifugation was performed to separate the nanoparticles from the initial solution. The amount of sorafenib associated with nanoparticles was determined by the change in the absorption spectrum of the solution before the addition of nanoparticles and after the deposition of nanoparticles. The procedure was carried out 3 times. The hydrodynamic radius of selenium nanoparticles increased after addition of sorafenib to it (Figure 9). The zeta potential increased to −23 mV. Data on microscopic examination of nanoparticles with adsorbed sorafenib molecules are not carried out, since the sorafenib coat has an extremely low contrast.

### 4.3. Cell Culture

Cell line HepG2 (Human Caucasian hepatocyte carcinoma) was purchased from ATCC (ATCC HB-8065, Manassas, VA, USA) and was grown on round coverslips for 48 h in a CO_2_-incubator in DMEM medium supplemented with 10% fetal bovine serum until a confluence of 80–95% was achieved. Cell cultures were used from the third passage. We checked our continuous cell culture for mycoplasma contamination every three month by PCR as recommended by Drexler and Uphoff [71]. No mycoplasma contamination was detected.

### 4.4. Assessment of Cell Viability and Apoptosis

A single treatment of cells was performed with concentrations of the studied compounds. Sorafenib was added to one experimental group, “naked” SeNPs were added to the other, and the selenium–sorafenib nanocomplex was added to the third. Cell survival experiments were performed after 24 h and 48 h of treatment. Cell death (apoptosis or necrosis processes) in the cell culture was assessed by simultaneous staining of cells with Propidium iodide (PI, 1 µM) and Hoechst 33342 (HO342, 1 µM). It is known that viable cells are not permeable to PI, while Hoechst 33342 penetrates through the plasma membrane, staining the chromatin. Cultured HepG2 cells were defined as apoptotic if the intensity of Hoechst 33342 fluorescence was 3–4 times higher compared to Hoechst 33342 fluorescence in healthy cells, indicating chromatin condensation, which can occur as a result of apoptosis induction. The differences between the early and late stages of apoptosis were determined by the intensity of Hoechst 33342 fluorescence, and at the later stages of apoptosis, cells begin to show insignificant membrane permeability for PI [72,73]. The fluorescence of the probes was registered with a fluorescent system based on an inverted fluorescent microscope Axio Observer Z1 equipped with a high-speed monochrome CCD-camera Hamamatsu ORCA-Flash 2.8. The Lambda DG-4 Plus illuminator (Sutter Instruments, Novato, CA, USA) was used as a Source of excitation. To excite and record fluorescence of the probes we used: Filter Set 01 with excitation filter BP 365/12, beam splitter FT395, emission filter LP 397; Filter Set 20 with excitation filter BP 546/12, beam splitter FT560, emission filter BP 575–640. We used objective HCX PL APO 20.0 × 0.70 IMM UV, refraction index 1.52. Camera settings is 500 pixels × 500 pixels (Voxel Size 0.724 µm × 0.723 µm), binning 2 × 2, resolution 14 bits. Five different fields of view were analyzed for each coverslip with cells. Each experiment was repeated three times with separate cell cultures.

### 4.5. MTT Assay

To assess cell viability, which makes it possible to estimate the number of living cells in the test sample and the rate of their proliferation, MTT assay was used using the MTT Cell Proliferation Assay Kit (Abcam, Cambridge, UK). To do this, cells were grown on 96-well plates up to approximately 5500 cells per well and incubation was carried out for 24 h with different concentrations of SeNPs or SeSo, after which 20 μL of MTT working solution (5 mg/mL) in Sodium phosphate buffer, pH 7.4, was added, carefully pipetted and incubated for 3 h at 37 °C and 5% CO_2_. Next, the medium was removed, and the cells were slightly dried. For fast and uniform dissolution of formazan, 200 µL of DMSO was added to each well, and incubated on a plate shaker for 10 min at room temperature. Next, we used a plate reader to measure the optical density of Solutions in each test well at OD = 570 nm and background values at OD = 670 nm. This procedure was repeated three times.

### 4.6. Fluorescent Ca^2+^ Measurements

Experiments were carried out in the daytime. The measurements of [Ca^2+^]_i_ were performed by fluorescence microscopy using Fura-2/AM. A-172 cells were loaded with the probe dissolved in Hanks balanced salt solution (HBSS) composed of (mM): 156 NaCl, 3 KCl, 2 MgSO_4_, 1.25 KH_2_PO_4_, 2 CaCl_2_, 10 glucose and 10 HEPES, pH 7.4 at 37°C for 40 min with subsequent 15 min washout. To measure free cytosolic Ca^2+^ concentration, we used the Carl Zeiss Cell Observer and an inverted motorized microscope Axiovert 200M with a high-speed monochrome CCD-camera AxioCam HSm with a high-speed light filter replacing system, Ludl MAC5000. Fura-2 excitation and registration were recorded using a 21HE filter set (Carl Zeiss, Oberkochen, Germany) with excitation filters BP340/30 and BP387/15, beam splitter FT-409 and emission filter BP510/90, objective lens HC PL Fluotar 10×/0.3 Dry, refraction index 1, and excitation light source HBO 103W/2. Camera settings is 500 pixels × 500 pixels (Voxel Size 0.724 µm × 0.723 µm), binning 2 × 2, resolution 14 bits [44]. Background fluorescence was subtracted frame by frame using the Math Subtract plugin in ImageJ. Calcium responses were shown as a ratio of Fura-2 fluorescence intensities. Therefore, we determined the amplitudes of Ca^2+^ responses to SeNPs as (Δ) − Fmax–Fmin of Fura-2 fluorescence and an increase in Fura-2 fluorescence reflects a linear [Ca^2+^]_i_ increase in response to receptor agonists. ImageJ 2002 Software (RRID: SCR_003070, NIH Image, Bethesda, MD, USA) was used to analyze data.

### 4.7. Extraction of RNA and Real-Time Polymerase Chain Reaction (RT-qPCR)

Total RNA from HepG2 cells after 24 h or 48 h of treatment with various concentrations of SeNP or SeSo was isolated using ExtractRNA reagent according to the manufacturer’s instructions (Evrogene, Moscow, Russia). The concentration and purity of the total RNA was determined spectrophotometrically at 260/280 nm. First-strand cDNA was synthesized from 1–2 µg of total RNA using MMLV reverse transcriptase according to the manufacturer’s instructions (Evrogene, Russia). Real-time qPCRs were performed in a 25 µL reaction mixture containing SYBR Green I PCR Master Mix (Evrogene, Russia) and 300 nM of the appropriate primers (Table 1). The PCR procedure consisted of 94°C for 2 min followed by 35 cycles of 94 °C for 1 min, 60 °C for 30 s, and 72 °C for 30 s. Glyceraldehyde-3-phosphate dehydrogenase (GAPDH) was used as an internal control for normalization, and results were expressed as 2^−∆(∆Ct) )^, where Δ(ΔCt).

### 4.8. Western Blotting

Cells were homogenized with Cell Lysis Buffer (100 mM Tris–HCl, pH 8.0, 0.15 mM NaCl, 1 mM EDTA, 1 mM PMSF). The lysates were cleared by centrifugation at 14,000× *g* for 10 min at 4 °C. Proteins were separated by SDS-PAGE on 12.5% polyacrylamide gels and were transferred onto nitrocellulose membrane and were blocked for 1 h at room temperature in 5% non-fat dry milk in 1× PBS. Nitrocellulose membranes were subsequently incubated overnight at 4 °C with primary antibodies (1:100–1:500). Thereafter, blots were incubated for 1 h with the secondary antibody conjugated to horseradish peroxidase (1:5000). Immunoreactive bands were visualized by detection of peroxidase activity by DAB staining (0.05% DAB in TBS + 10 μL 30% hydrogen peroxide). The analysis of the obtained results was carried out using the ImageJ 2002 Software and Analyze plug-ins (Gels). The following primary and secondary commercial antibodies purchased from Abcam (Cambridge, UK) and Invitrogene (Waltham, MA, USA) were used in the work: anti-GAPDH (#437000, Invitrogen), anti-SELENOM (#PA5-72639, Invitrogen), anti-SELENOT (#PA5-26314, Invitrogen), anti-SELENON (#PA5-43082, Invitrogen), anti-DIO2 (#PA5-100474, Invitrogen), anti CAS-4 (#PA5-118880, Invitrogen), anti BAX (#33-6400, Invitrogen) and secondary, conjugated with horseradish peroxidase (#656120, Invitrogen).

### 4.9. Statistical Analysis

All presented data were obtained from at least three cell cultures from 2–3 different passages. All values are given as mean ± standard error (SEM) or typical Ca^2+^-signals. Statistical analyses were performed by paired t-test. ImageJ, Origin 2016 (OriginLab, Northampton, MA, USA), and Prism GraphPad 7 (GraphPad Software, La Jolla, CA, USA, RRID: SCR_002798). Software was used for data and statistical analysis.

## 5. Conclusions

Thus, the study made it possible to establish the mechanisms of the complex anticancer effect of selenium nanoparticles and the selenium–sorafenib nanocomplex on HepG2 cells. Dominant signaling pathways were identified that determine the advantage of the selenium–sorafenib nanocomplex in the induction of apoptosis, compared with selenium nanoparticles and sorafenib. Nanoselenium and the selenium–sorafenib nanocomplex have a significantly more pronounced anticancer effect compared to sorafenib. The full synergistic effect with the use of the nanocomplex selenium–sorafenib was not observed after 24 h of cell treatment, but the effect was significantly enhanced after 48 h, compared with naked nanoparticles. It is likely that the selenium–sorafenib nanocomplex takes longer to release selenium into the cell after endocytosis of SeSo into the cell. At the same time, the selenium–sorafenib nanocomplex provides targeting in relation to liver cancer and contributes to a more effective and prolonged activation of the regulated death of cancer cells.

## Figures and Tables

**Table 1:** Primers for real-time PCR.

Gene Name	Forward Primer 5′→3′	Reverse Primer 5′→3′
*GAPDH*	ACATCGCTCAGACACCATG	GCCAGTGAGCTTCCCGTT
*SELENOT*	TCTCCTAGTGGCGGCGTC	GTCTATATATTGGTTGAGGGAGG
*SELENOM*	AGCCTCCTGTTGCCTCCGC	AGGTCAGCGTGGTCCGAAG
*SELENOF*	TACGGTTGTTGTTGGCGAC	CAAATTGTGCTTCCTCCTGAC
*SELENOK*	TTTACATCTCGAACGGACAAG	CAGCCTTCCACTTCTTGATG
*SELENOS*	TGGGACAGCATGCAAGAAG	GCGTCCAGGTCTCCAGG
*SELENON*	TGATCTGCCTGCCCAATG	TCAGGAACTGCATGTAGGTGG
*DIO2*	AGCTTCCTCCTCGATGCC	AAAGGAGGTCAAGTGGCTG
*GPX1*	CTACTTATCGAGAATGTGGCG	CGAAGAGCATGAAGTTGGG
*GPX4*	CCATGCACGAGTTTTCCG	AATTTGACGTTGTAGCCCG
*TXNRD1*	GGTCTGGCAGCTGCTAAGG	TAGCCCCAATTCAAAGAGC
*TXNRD3*	CCTTTGCTTTGTTGTTTCTGTG	TAGTGAGTGTGAGGGTGAAGC
*CHOP*	GCTCTGATTGACCGAATGG	TCTGGGAAAGGTGGGTAGTG
*GADD34*	CTCCGAGAAGGTCACTGTCC	GACGAGCGGGAAGGTGTGG
*PUMA*	CAGATATGCGCCCAGAGAT	CCATTCGTGGGTGGTCTTC
*BIM*	GGACGACCTCAACGCACAGTACGAG	GTAAGGGCAGGAGTCCCA
*ATF–4*	GTGTTCTCTGTGGGTCTGCC	GACCCTTTTCTTCCCCCTTG
*ATF–6*	AACCCTAGTGTGAGCCCTGC	GTTCAGAGCACCCTGAAGA
*XBPu*	ACTCAGACTACGTGCACCTC	GTCAATACCGCCAGAATCC
*XBPs*	CTGAGTCCGCAGCGGTGCAGG	GGTCCAAGTTGTCCAGAATG
*CAS–3*	GCATTGAGACAGACAGTGGTG	AATAGAGTTCTTTTGTGAGCATG
*CAS–4*	CACGCCTGGCTCTCATCATA	TAGCAAATGCCCTCAGCG
*BAX*	GGGCTGGACATTGGACTTC	AACACAGTCCAAGGCAGCTG
*BAK*	GAGAGTGGCATCAATTGGGG	CAGCCACCCCTCTGTGCAATCCA
*BCL-2*	GGTGAACTGGGGGAGGATTG	AGCCAGGAGAAATCAAACAGAG
*RIPK1*	GCCATTCAGCTCCTTGCCAC	CAGTTTACGGGCACAGTTTTTC
*RIPK3*	AGCCCTACCTCAACTGGAAC	CCAGGCTTCAGGATCTTTAGG
*MLKL*	TGAGAAGATCCGCAAGCTGG	TTTGTGCCTCTCCCAGCTTC
*RAS*	AACAAGTGTGACCTGGCTGC	TCCGGCACCTCCATGTCCTG
*RAF*	CCCCAAAGCAATGAAGAGGC	TCAACTAGAAGACAGGCAGCC
*MAPK1*	AGCAGTATTACGACCCGAGTG	CTGGGAAGAAGAACACCGATG
*MAPK3*	GCTGGCTCACCCCTACCTG	ATTTTCTAACAGTCTGGCGGG
*PI3K*	CAAATACATTAGGAGCGAAGGC	CAAATACATTAGGAGCGAAGGC
*mTOR*	TTACGACGGCATGGGAATCTC	CAAATACATTAGGAGCGAAGGC
*KIT*	AACGCTCGACTACCTGTGAAG	GCTTGAATGTTGGTCTTTTTAGG
*FLT3*	AGAAGCGATGTATCAGAATGTG	GTGAAGCAGCAGTTGATAATAG
*VEGFR*	CTGACTCGGAAGGACACGG	CCTGGCGGCACGAAATATCC

## Data Availability

The data presented in this study are available on request from the corresponding author.

## Supplementary Materials

The following supporting information can be downloaded at: https://www.mdpi.com/article/10.3390/ijms23126641/s1.
