# Supplementary material for: Comparative Analysis of the Cytotoxic Effect of a Complex of Selenium Nanoparticles Doped with Sorafenib, “Naked” Selenium Nanoparticles, and Sorafenib on Human Hepatocyte Carcinoma HepG2 Cells
Source: Int J Mol Sci. 2022 Jun 14;23(12):6641. doi: 10.3390/ijms23126641 (PMC9223423; doi:10.3390/ijms23126641)
Supplement: Supplementary file 1 [file ijms-23-06641-s001.zip › ijms-1746100-supplementary.pdf]

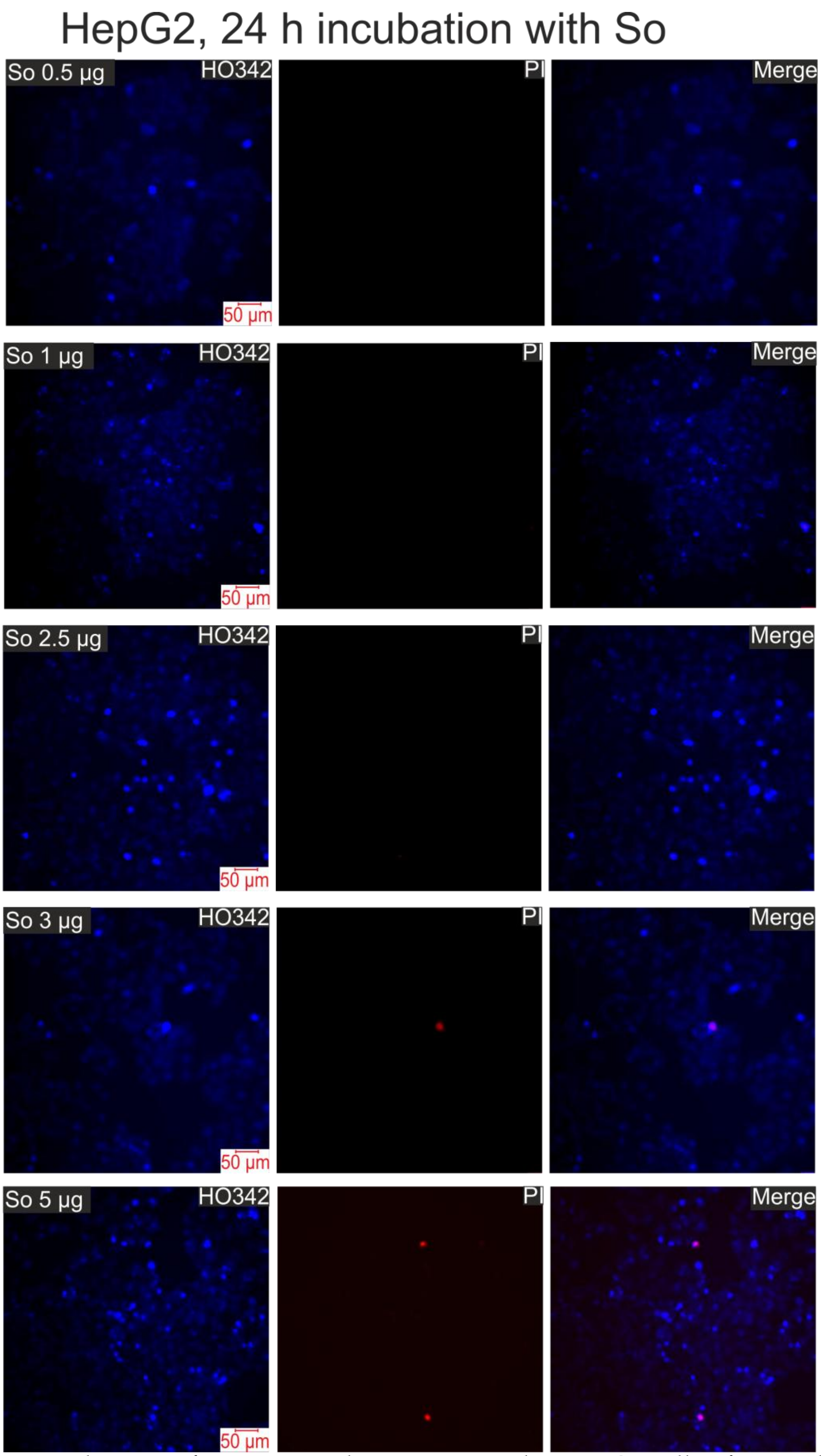

FIGURE S1. Induction of necrosis and apoptosis in the HepG2 cells after 24 hours of incubation with various concentrations of sorafenib (So), depending on the preliminary incubation with 0.5, 1, 2.5, 3 and 5 µg/ml So. Double staining of cells with Hoechst 33342 (HO342), Propidium iodide (PI) and merge (Merge). The images shown in the figure correspond to the data in Figure 2 of the text of the manuscript.

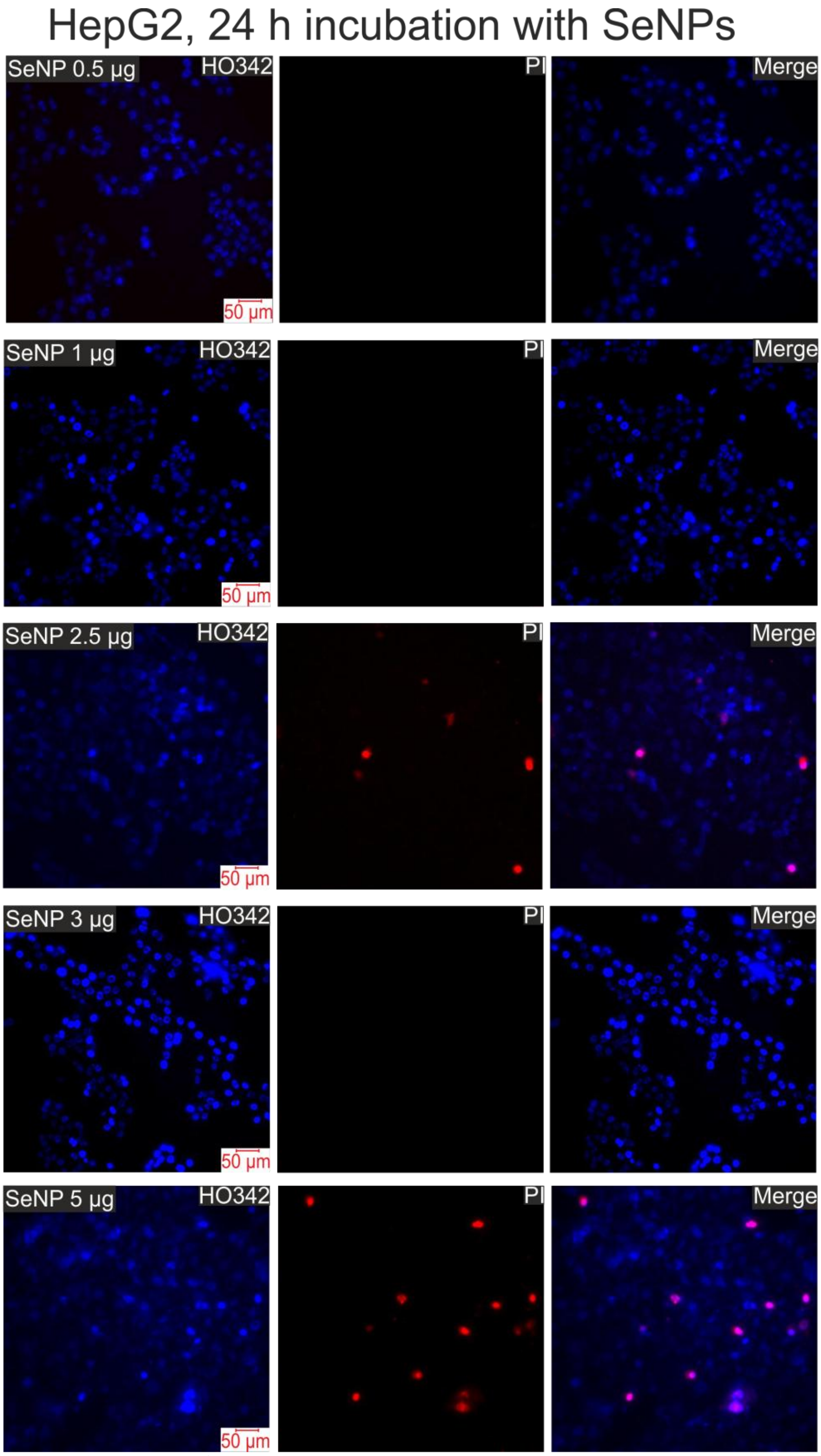

FIGURE S2. Induction of necrosis and apoptosis in the HepG2 cells after 24 h incubation with various concentrations of 50 nm selenium nanoparticles (SeNPs), depending on the preliminary incubation with 0.5, 1, 2.5, 3 and 5 µg/ml SeNPs. Double staining of cells with Hoechst 33342 (HO342), Propidium iodide (PI) and merge (Merge). The images shown in the figure correspond to the data in figure 2 of the text of the manuscript.

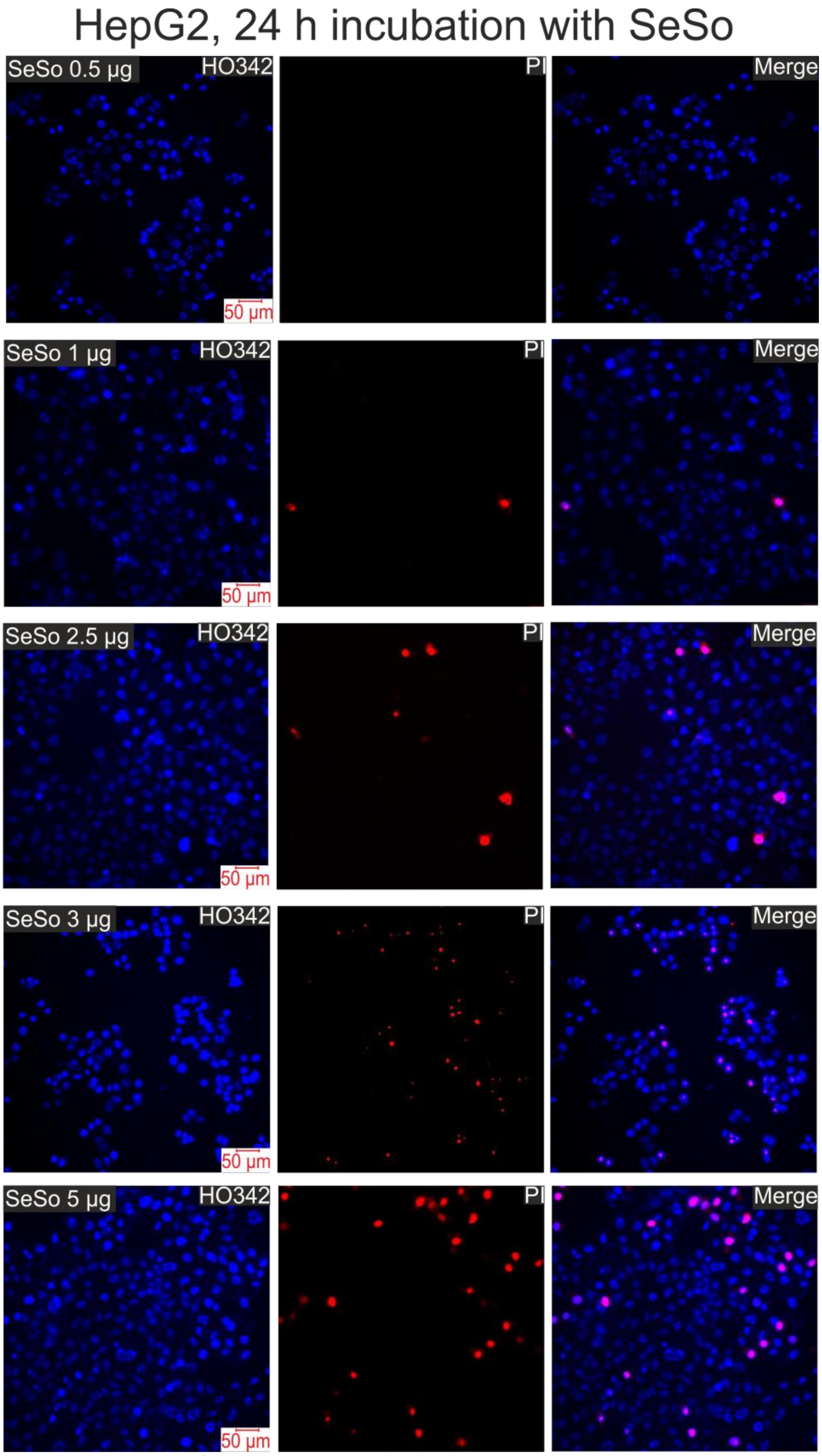

FIGURE S3. Induction of necrosis and apoptosis in the HepG2 cells after 24 h incubation with various concentrations of 50 nm selenium nanoparticles doped with sorafenib (SeSo), depending on the preliminary incubation with 0.5, 1, 2.5, 3 and 5 µg/ml SeSo. Double staining of cells with Hoechst 33342 (HO342), Propidium iodide (PI) and merge (Merge). The images shown in the figure correspond to the data in figure 2 of the text of the manuscript.

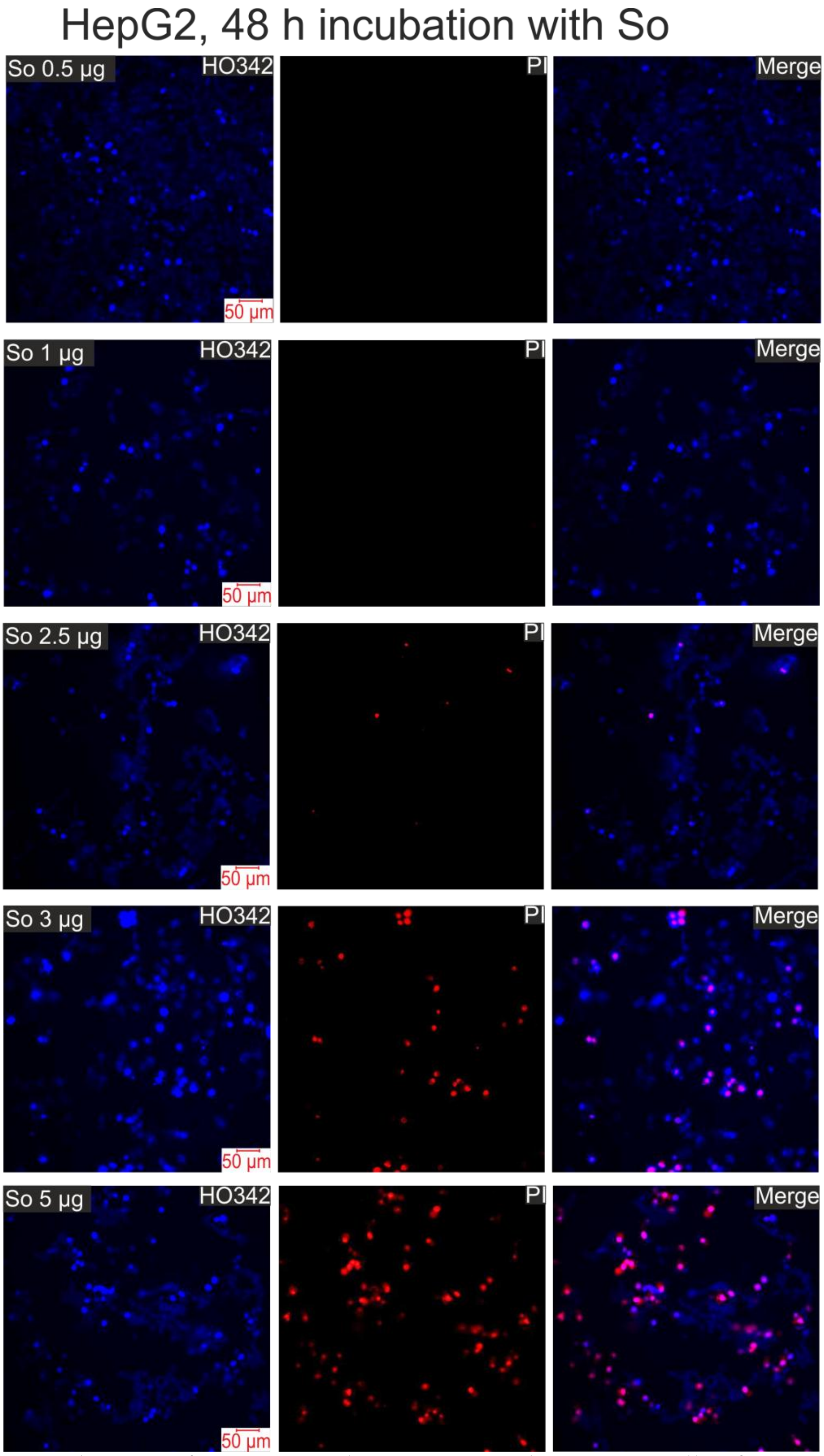

FIGURE S4. Induction of necrosis and apoptosis in the HepG2 cells after 48 hours of incubation with various concentrations of sorafenib (So), depending on the preliminary incubation with 0.5, 1, 2.5, 3 and 5 µg/ml So. Double staining of cells with Hoechst 33342 (HO342), Propidium iodide (PI) and merge (Merge). The images shown in the figure correspond to the data in figure 3 of the text of the manuscript.

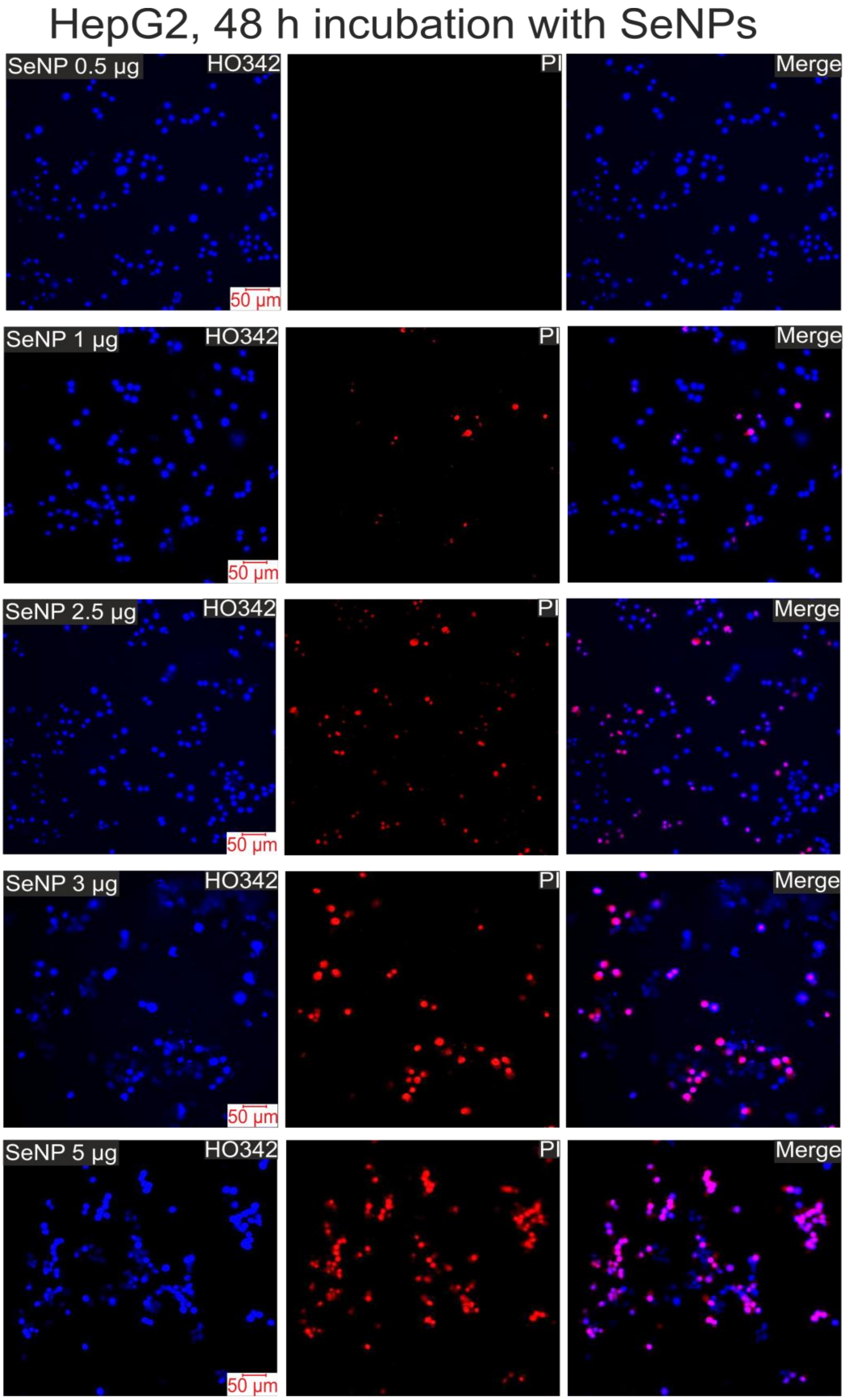

FIGURE S5. Induction of necrosis and apoptosis in the HepG2 cells after 48 h incubation with various concentrations of 50 nm selenium nanoparticles (SeNPs), depending on the preliminary incubation with 0.5, 1, 2.5, 3 and 5 µg/ml SeNPs. Double staining of cells with Hoechst 33342 (HO342), Propidium iodide (PI) and merge (Merge). The images shown in the figure correspond to the data in figure 3 of the text of the manuscript.

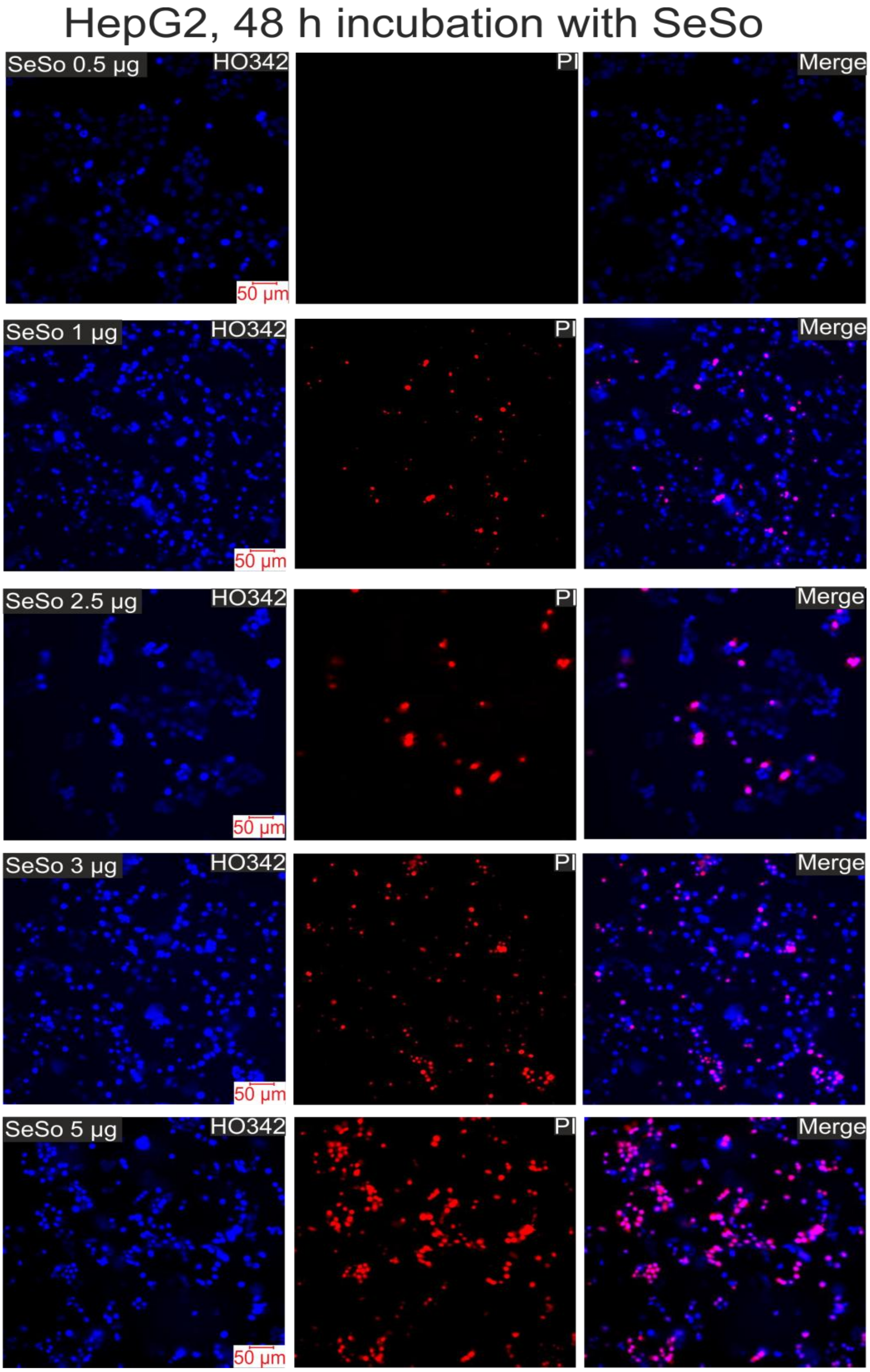

FIGURE S6. Induction of necrosis and apoptosis in the HepG2 cells after 48 h incubation with various concentrations of 50 nm selenium nanoparticles doped with sorafenib (SeSo), depending on the preliminary incubation with 0.5, 1, 2.5, 3 and 5 µg/ml SeSo. Double staining of cells with Hoechst 33342 (HO342), Propidium iodide (PI) and merge (Merge). The images shown in the figure correspond to the data in figure 3 of the text of the manuscript.

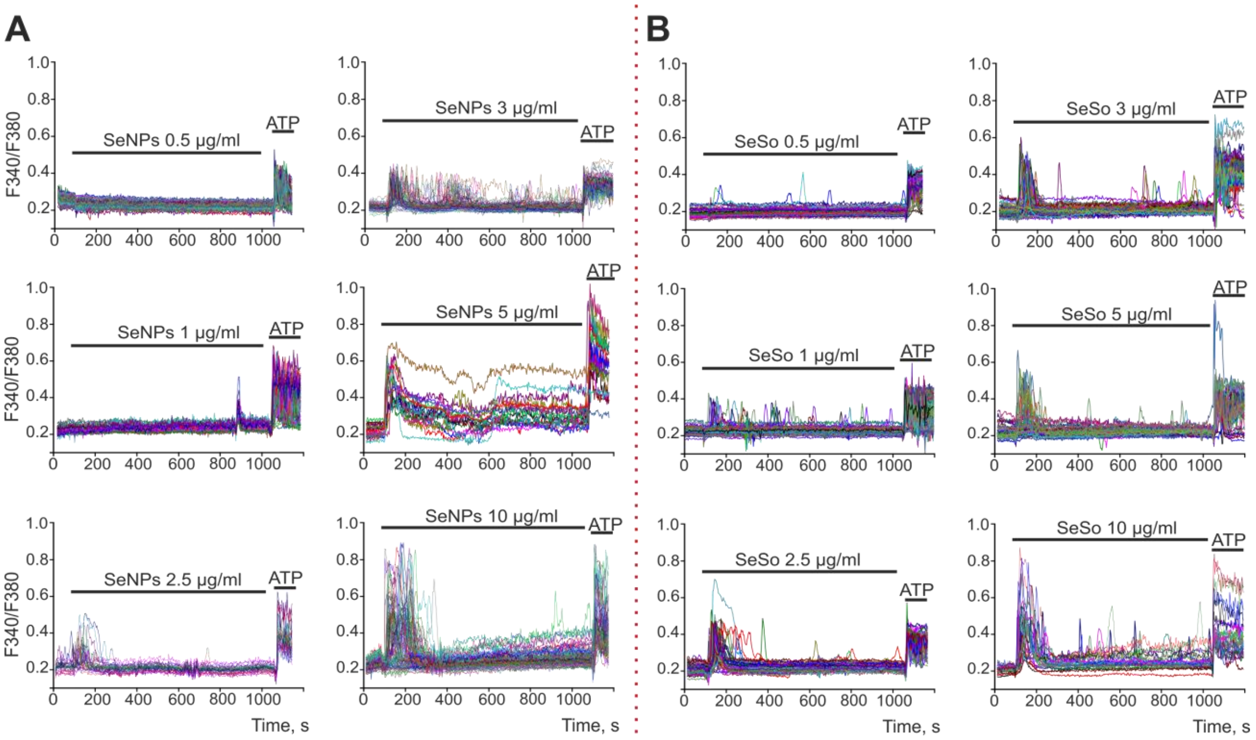

FIGURE S7. Ca^{2+} responses of HepG2 cells to the application of various concentrations of SeNPs (**A**) and SeSo (**B**). Ca^{2+} signals in one experiment are shown. At the end of the experiments, 10 μM ATP was applicated.

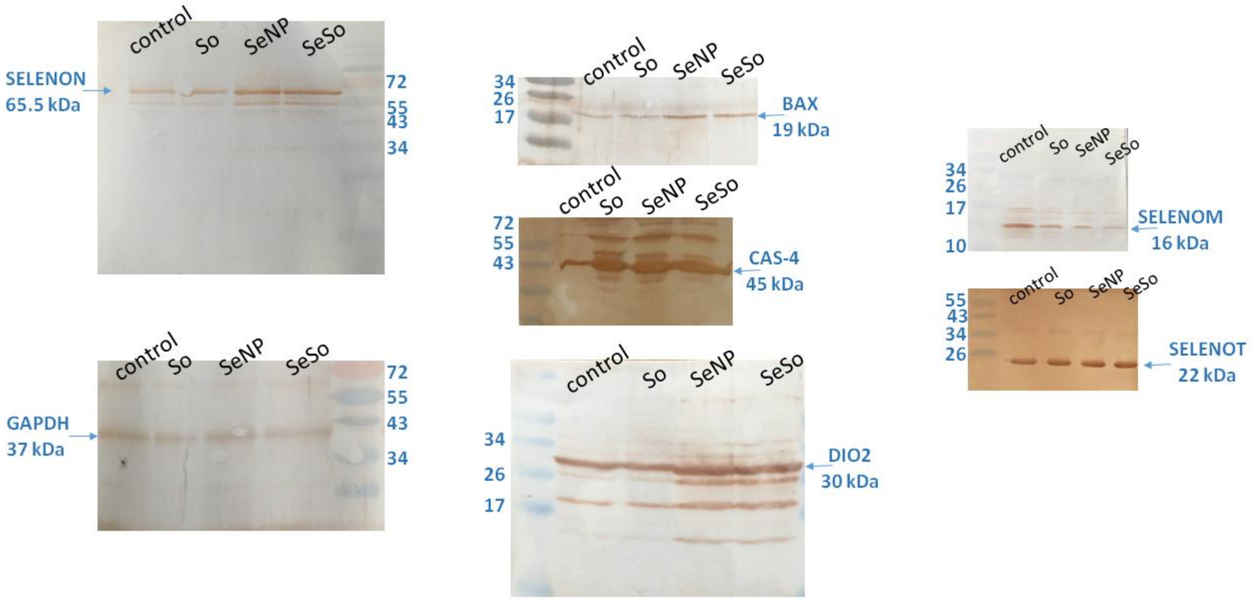

FIGURE S8. Western blots (original) of selenoproteins and pro-apoptotic proteins content in HepG2 cells after 48 h treatment with 3 μg/ml So, SeNPs, and SeSo.
